# Outcomes Following Treatment with Notched Proton Beams for Peripapillary Choroidal Melanomas

**DOI:** 10.3390/cancers17223684

**Published:** 2025-11-18

**Authors:** Gulmeena Hussain, Jonathan Lam, Antonio Eleuteri, Linda Mortimer, Andrzej Kacperek, Bertil Damato, Heinrich Heimann, Rumana Hussain

**Affiliations:** 1 Liverpool Ocular Oncology Centre, St Paul’s Eye Unit, Royal Liverpool University Hospital, Liverpool L7 8XP, UK; 2 Department of Medical Physics and Clinical Engineering, Royal Liverpool University Hospital, Prescot St., Liverpool L7 8XP, UK; 3 National Eye Proton Therapy Centre, The Clatterbridge Cancer Centre, Wirral CH63 4JY, UK

**Keywords:** ophthalmology, proton beam therapy, choroidal melanoma, uveal melanoma, visual acuity

## Abstract

Purpose: Peripapillary choroidal melanoma provides a unique challenge; proximity to visually important structures, such as the optic disc and fovea, confers a high risk for the development of maculopathy and optic neuropathy, leading to poorer visual outcomes with most forms of radiotherapy. Ocular proton therapy (OPT) requires an aperture to shape the beam to the tumour. An aperture ‘notch’ may minimise damage to the optic disc and/or the fovea. This study aims to explore if there are any additional advantages to incorporating a notch over the optic nerve beam area. Design: Retrospective audit (cohort study). Participants: Participants included eighty-three patients treated at Liverpool with proton beam therapy from January 2012 to March 2020 for their peripapillary choroidal melanoma. All had a minimum of two and a half years of follow-up vision data; this was to ensure there was enough visual acuity assessment data to perform sufficient analysis. Patients excluded had choroidal melanoma situated over 3 mm from the optic disc, as these were unlikely to have an aperture notch. Methods: A retrospective audit was undertaken in accordance with the Declaration of Helsinki, and registered with the Royal Liverpool Hospitals audit department (audit reference number: Ophth/SE/2024-25/25). Data was collated from the Liverpool Ocular Oncology database, clinic letters and the individual proton beam 3D plans. Robust statistical analysis using a mixed effects model was used to explore associations between notched beams and vision loss and complications. Main Outcome Measures: The primary outcome measure is visual acuity loss post-proton beam therapy. Secondary outcome measures were enucleation and other complication rates. Results: Analysis shows that at 10 years post-OPT, there would be an expected 0.058 (*p* = 0.077) logMAR of vision saved using a notch for the optic disc compared to no notch (normal apertures); this is considered clinically significant. This cohort also loses vision at a slower rate than other cases. No other predictors were found to be statistically significant for loss of vision, and notched beams showed no advantage in reducing rates of complications. Conclusions: There is some evidence of a trend that utilising a notch for optic disc does show long-term vision benefit; it demonstrates a clinically significant benefit in patients with peripapillary choroidal melanoma.

## Simple Summary

Peripapillary choroidal melanoma provides a unique challenge; proximity to visually important structures, such as the optic disc and fovea, confers a high risk for the development of maculopathy and optic neuropathy, leading to poorer visual outcomes with most forms of radiotherapy. Ocular proton therapy (OPT) requires an aperture to shape the beam to the tumour. An aperture ‘notch’ may minimise damage to the optic disc and/or the fovea. This study aims to explore if there are any additional advantages to incorporating a notch over the optic nerve beam area. The primary outcomes were longitudinal measurements of visual acuity following proton beam radiotherapy, the occurrence of radiation optic neuropathy, and the tumour recurrence rate. Secondary outcome measures included mortality rates, enucleation rate, and other potentially vision-affecting complications such as radiation maculopathy. Our findings demonstrate that notched proton beam therapy may offer a clinically meaningful reduction in long-term vision loss for tumours located within 3 mm of the optic disc. While not statistically significant, the trend toward slower visual decline and lower complication rates—especially when optic nerve exposure is minimised—supports consideration of this approach in treatment planning. Further validation in prospective, multi-centre studies is warranted.

## 1. Introduction

Choroidal melanoma (CM) is the most common primary adult intraocular malignancy (accounting for 90% of uveal melanoma) [1,2]. It affects approximately 700 to 800 patients annually in the UK [1,3], who are predominantly Caucasian [4,5]. Unfortunately, despite the treatment options available, 30–40% of patients with this disease will die from metastatic disease (notably to the liver) [6,7]. Most CMs are treated with radiotherapy, and in the UK, there are three mainstay forms of radiotherapy: ocular proton therapy (OPT); brachytherapy using ruthenium-106 plaque and stereotactic therapy (SRT) [8]. 20-40%% of UK patients with uveal melanoma were treated with OPT [9].; Liverpool Ocular Oncology Centre is one of four in the UK to offer this, and the Clatterbridge eye proton therapy service is accessible to UK residents [10].

Treating peripapillary melanomas (near the optic disc), juxtapapillary melanomas (touching the optic disc), and circumpapillary melanomas (surrounding or on the optic disc) presents unique challenges. The optic nerve and its sheath pose significant obstacles for plaque placement, with the sheath potentially measuring up to 8 mm in diameter, restricting plaque advancement. The high recurrence rates and treatment failures associated with these melanomas are primarily due to inadequate local brachytherapy radiation reaching the tumour effectively, near the optic nerve. Additionally, impingement of the optic nerve when placing the plaque may result in arterial occlusion to the nerve and its loss of blood supply [11], resulting in permanent and irreversible vision loss.

To address the challenges associated with plaque advancement beyond the optic nerve sheath, stereotactic radiotherapy may be used. This technique utilises a precisely focused beam of radiation directed at the tumour margin and tailored to the appropriate depth within the eye. One effective modality is OPT, which has demonstrated the lowest recurrence rates amongst other forms of stereotactic radiotherapy, in the treatment of choroidal melanoma [12,13].

Proton therapy capitalises on the Bragg peak effect, wherein the distal radiation dose is controllable and falls off very sharply [14,15]; additionally there is minimal scatter of radiation compared to conventional x-ray techniques [16].

**Figure 1:**
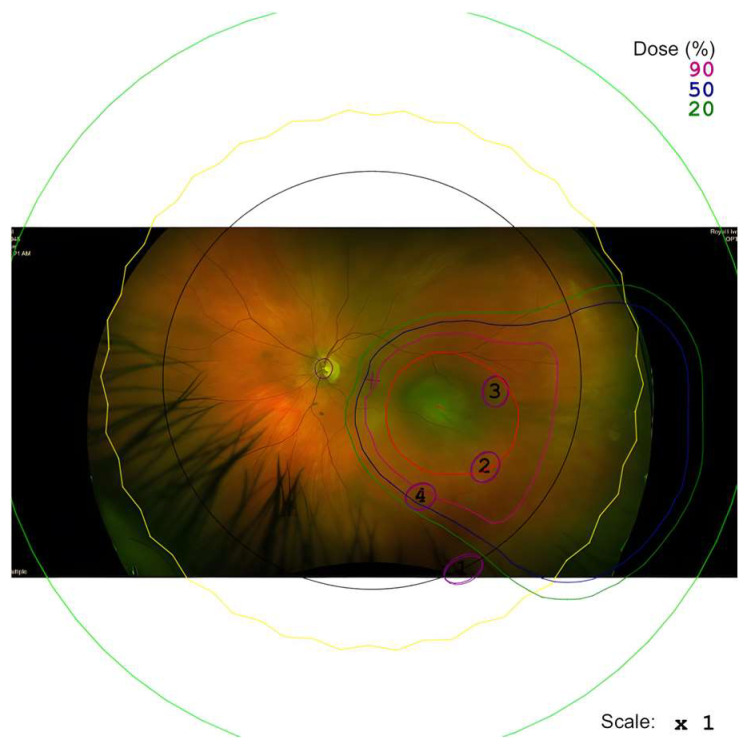
An example of a proton beam plan for the tumour is seen with an optic disc notch added in the plan.

This specific property allows for a detailed model of the treatment area can be created (as seen in Figure 1) to avoid delivering high radiation doses to critical structures within the eye, such as the optic nerve. For peripapillary tumours located near the optic nerve, a ‘notched’ proton beam can be modelled, where the treatment area encompassing the choroidal melanoma is notched to exclude the optic nerve head. A sufficient treatment margin may be established without administering the complete dose to the optic nerve. We hypothesise that this approach may help to preserve the optic nerve’s blood supply and minimise the risk of complications, such as radiation-induced optic neuropathy, whilst still being sufficient in treating the entire choroidal melanoma.

This study seeks to examine whether employing a proton beam notched around the optic disc and/or fovea in patients with peripapillary choroidal melanoma offers any benefits for visual outcomes. Additionally, it aims to assess whether the use of a notch affects treatment efficacy.

## 2. Methods

A retrospective audit was undertaken in accordance with the Declaration of Helsinki, and registered with the Royal Liverpool Hospitals audit department (audit reference number: Ophth/SE/2024-25/25). All patients sign an informed consent for audit and research. We compare all patients with peripapillary choroidal melanoma from January 2012 to May 2019 who underwent OPT at the Liverpool Oncology Centre looking at visual outcomes for patients treated with a notched beam for optic disc, versus an un-notched beam. Peripapillary choroidal melanomas were defined as being within 3 mm of the optic disc, including touching the optic disc.

Patients excluded were those with less than two and a half years’ total follow-up vision data (given that vision-affecting radiation complications, such as radiation optic neuropathy and maculopathy, may have a delayed onset, and to allow for any meaningful trends to be delineated). The study includes adult participants only.

The primary outcomes were longitudinal measurements of visual acuity following proton beam radiotherapy, the occurrence of radiation optic neuropathy, and the tumour recurrence rate. Secondary outcome measures included mortality rates, enucleation rate, and other potentially vision-affecting complications such as radiation maculopathy. A retrospective analysis of the Royal Liverpool University Hospital database was undertaken to collect this data. Fundus photography was used to measure the distance of the choroidal melanoma to the disc (that being the nearest point of the melanoma, measured to the disc edge).

Proton beam treatments were modelled using EYEPLAN software (V. 3.). Treatments were modelled with either a notch to the optic nerve head or no notch, and retrospective analysis of the plans was performed to group patients as notched or un-notched. The EYEPLAN treatment plans were created in collaboration with the Clatterbridge radio physicists. If it was considered that a notched plan may protect some vision without compromising tumour control, this was preferred.

Additional data such as the length of the optic nerve irradiated with the treatment dose of 53 Gy was also collected from these plans. 52 Gy is the (total) physical radiation dose given to the patient [17]. The cobalt-60 equivalent dose is 10% higher due to a biological effectiveness of 10% (i.e., RBE = 1.1). Therefore, the total Gy(E) or Gy(RBE) would be 57.2 Gy(RBE).

### Linear Regression Model Analysis

To account for longitudinal observations with an uneven number of follow-up visits, an extended linear regression model [18] was fitted. Analyses were implemented in R (v. 4.2.3) code analysis, using the *rms* and *nlme* libraries. The model allowed statistical inference of the effects of baseline predictors on visual outcomes:

Predictor variables of visual acuity, identified for each subject included: age, sex, tumour volume, tumour stage, distance of tumour from optic disc, proton beam notch type, radiation retinopathy indicator, optic neuropathy indicator, vitreous haemorrhage indicator, radiation maculopathy indicator, length of optic nerve treated, follow-up years, and interaction between follow-up years and proton beam notch type.

To perform inference, an extended linear regression model was fitted. The model can be written in matrix notation as follows:

$$y_{i}=X_{i}\beta +\epsilon_{i}\;$$

$$\epsilon_{i}\sim N\left(0,\sigma^{2}\Lambda_{i}\right)\;,\;i=1,\ldots \;,84$$

where for the $i^{th}$ subject,

- $y_{i}$ is the vector of longitudinal relative logMAR observations;

- $X_{i}\;$ is the regressor matrix;

- $\epsilon_{i}\;$ is the vector of Gaussian within-group errors with variance–covariance matrix $\sigma^{2}\Lambda_{i}$;

- β  is the vector of coefficients of the model.

**Figure 2:**
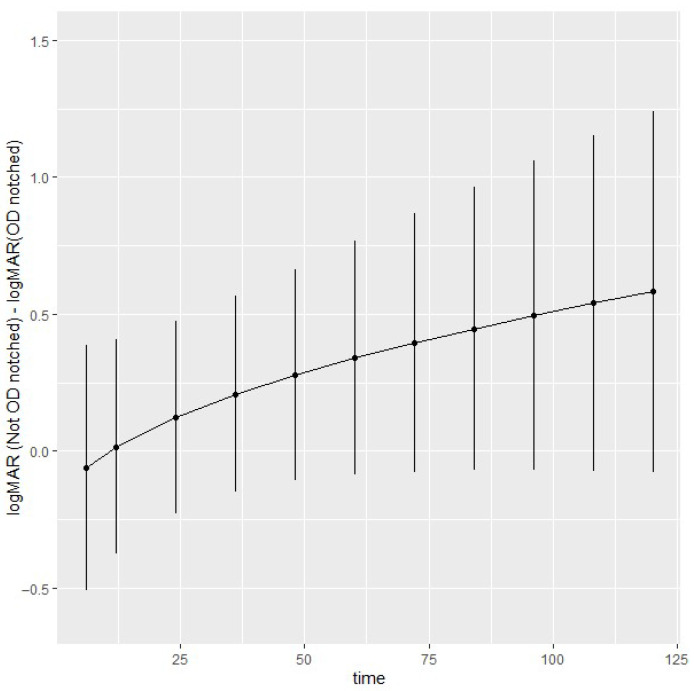
Estimated optic disc notch contrasts at 6 months, and at 1-year intervals up to 10 years of follow-up. Time on the *x*-axis refers to months.

To model the nonlinearity of the trajectories observed in Figure 2, the follow-up years have been transformed by taking the square root (though the results in the following graphs will be presented on the “natural years” time scale). The variance–covariance matrix models the observed heteroscedasticity and dependence among measurements of relative logMAR. Model diagnostics and estimates of the variance–covariance terms are reported in Appendix A.

## 3. Results

### 3.1. Baseline Characteristics

The data set is composed of 464 observations on 83 subjects. A summary of the patient characteristics and tumour characteristics can be found in Table 1. The minimum follow-up time was three months, and the maximum was 118 months. For each subject, baseline characteristics at treatment were analysed: logMAR, age, sex, tumour volume, tumour stage, distance of tumour from fovea, distance of tumour from optic disc, proton beam notch type, and length of optic nerve irradiated. The median age of this cohort was 59.2 years, and 65.0% were male. Average tumour diameter and height were roughly the same, comparing both groups of patients with notched or un-notched beams. The mean tumour volume had a wider range, from 2.5 to 802.4 mm^3^. The majority of tumours included were at stage T2a (59.5%). 20.2% of the cohort had tumours within 1 mm of the optic disc.

All patients received a proton beam dose of 53 Gy in four fractionated doses. Of this cohort, 56% had a notch for the optic disc and 54% had no notch at all. Mean follow-up time was 57.29 months (range from 30 to 118 months).

### 3.2. Predictors of Visual Acuity

The Wald statistics (as seen summarised in Table 2) show no strong evidence of association of logMAR with any of the predictors except follow-up years and baseline logMAR, with small evidence of an interaction effect between follow-up years and optic disc notch. Model validation results are reported in Appendix A.

**Figure A2:**
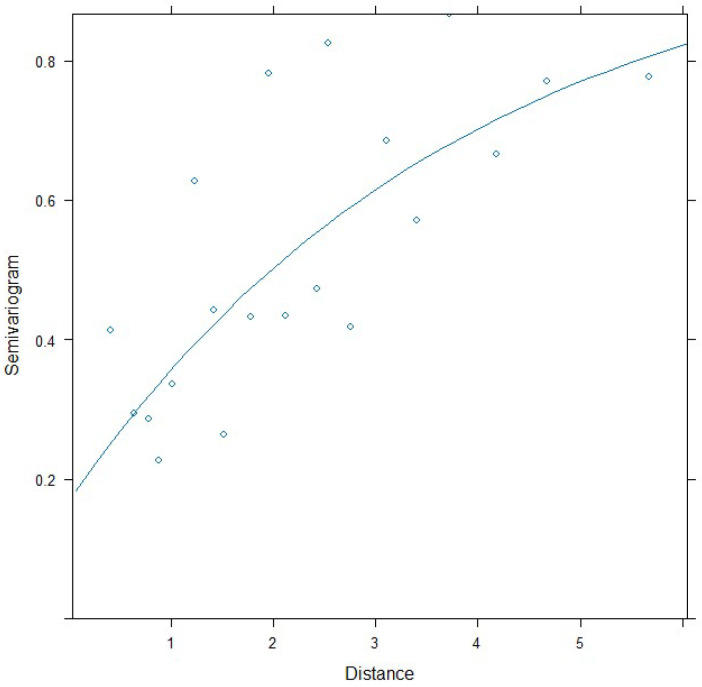
Variogram with assumed correlation pattern superimposed.

To explore the longitudinal effects of optic disc notch on logMAR, contrasts were estimated and are shown in Figure 2 with 95% simultaneous confidence intervals. At 10 years, the logMAR difference without and with optic disc notch is 0.58 [−0.077, 1.2]. Overall, there is some evidence of a trend of higher logMAR without optic disc notch compared to optic disc notch; however, the small number of observations in later years produces wider confidence intervals. The contrasts reflect the expected trends in Figure A2, available in Appendix A.

### 3.3. Speed of Vision Loss

Loss of visual acuity was seen to be slower in the notched group, versus the un-notched group. Trends and contrasts of relative visual acuity loss were plotted in both groups, with a 95% confidence interval. All slopes are positive (indicating an overall loss of vision over time) and significantly different from zero at the 95% level. The slowest loss is in the notched group with a slope of 0.19 versus 0.29 in the notched group (Table 3).

Contrast analysis of relative visual acuity (Table 4) demonstrated that at 10 years post-OPT, there would be on average about 0.57 (*p* = 0.076) logMAR of vision saved using a notch for the optic disc compared to no notch; this is considered of clinical significance.

### 3.4. Tumour Control

Recurrence occurred in 4.3% of patients with un-notched beams and in 3.1% in those with notched beams. Overall, the rate of recurrence was 3.8%. A summary of the secondary outcomes can be seen summarised in Table 5.

## 4. Optic Neuropathy

Optic neuropathy rates were 40.4% (n = 34) overall, with the highest frequency in the un-notched cohort (n = 13, 40.6%) and lowest in the notched group (n = 5, 15.6%).

No significant association between optic neuropathy and the presence of optic disc notch, or length of the optic nerve receiving the full proton beam treatment dose, or distance of the tumour from the optic disc, was determined. A logistic regression model was fitted to each of the 50 imputed data sets. In Table 6 are reported the Wald statistics and odds ratios.

A separate logistic regression model with a heteroscedastic-robust covariance matrix [18] (used to address the small event rate and potential data irregularities) did demonstrate a statistically significant association between the length of optic nerve treated and incidence of optic neuropathy (χ^2^ = 7.7, *p* = 0.006). The odds ratio was 3.1 [1.4, 6.9], indicating an increased risk of optic neuropathy with increasing nerve length irradiated.

The following optimism-corrected (via bootstrap, 1000 repetitions) validation measures were estimated: the rank discrimination is 0.74, the Brier score is 0.11, and the g-index (representing the “typical” odds ratio) is 2.3. Further model validation results are reported in Appendix B.

All the analyses were implemented in R v. 4.4.2, using the rms and nlme libraries.

### 4.1. Other Complications of Treatment

Complications of treatment (aside from optic neuropathy) were not found to be statistically significant predictors of relative visual acuity outcomes in either group (Table 5).

The rates of other complications are summarised in Table 5.

### 4.2. Metastasis

Metastasis occurred in 3.8% overall (n = 3). Two patients did not have a notch and one had a notch for the optic disc

## 5. Discussion

In this study, we evaluated the use of a notched versus un-notched proton beam to the optic nerve head, for the treatment of peripapillary choroidal melanoma, focusing on long-term visual acuity and complication outcomes. This represents the first cohort analysis to assess the clinical impact of customised notching around the optic disc.

Regarding treatment efficacy, the results did not show any significant difference in recurrence rate between the two treatment groups and have a very low overall tumour recurrence rate (0–4.3%), which is in line with what previous studies treating peri-papillary melanoma with OPT have shown. These rates are far better than those for notched plaque therapy reported at 2–32.5% [19,20,21], SRT at 6–7% [22,23,24], and PDT reported at 9% [25].

In other literature, enucleation rates may also be indicative of treatment failure to control choroidal melanoma, and reported rates vary significantly amongst different treatment modalities for peripapillary choroidal melanoma. Those reported for OPT range from 3.5 to 36.1% [26,27], and with plaque therapy from 4.3% [28] to the 87.5% reported by Sobti et al. in 2021 [20]. In this cohort study, the overall enucleation rate was 5.5%. Of those that underwent enucleation, 40% had an optic disc notch and 40% were un-notched, indicating that a notched treatment does not have an impact on the rate of enucleation; other factors such as stage of tumour or tumour volume are more likely to be influential predictors of enucleation rates.

The effect of notched versus un-notched treatment on visual acuity indicated that overall, vision loss occurred in both cohorts, however, less quickly in the notched group (slope of the trend of VA loss being 0.19 versus 0.29 in the notched and un-notched group, respectively). There is, however, a trend for long term vision benefit in using a notch for the optic disc (analysis shows at 10 years post-OPT, there would be an expected 0.57 (*p* = 0.076) logMAR of vision (approximately 20/80 Snellen equivalent) saved using a notch for the optic disc compared to no notch), in keeping with our study hypothesis.

While overall rates of optic neuropathy were notably higher in the un-notched cohort (40.6%) compared to the notched group (15.6%), the logistic regression model applied to the full data set did not find statistically significant associations between optic neuropathy and the presence of a notch or the distance of the tumour from the optic disc. However, a heteroscedastic-robust logistic regression model, employed as a sensitivity analysis to address limitations of small sample size and data irregularities, did show a statistically significant association between the length of optic nerve treated and the incidence of optic neuropathy (χ^2^ = 7.7, *p* = 0.006) with an odds ratio of 3.1 [1.4, 6.9]. This would suggest that per millimetre of optic nerve treated, the odds of developing visual loss from optic neuropathy multiply three-fold.

Other predictors of vision loss, including radiation maculopathy, did not show any statistical significance in our regression model. Cases of radiation maculopathy received anti-VEGF treatment in a treat-and-extend protocol to mitigate the visual effects of the complication [29,30].

It is important to note the limitations of this study, namely the sample size and bias that occurs with all retrospective analyses. There were not enough events of enucleation and recurrence to perform a full statistical analysis. This is the first retrospective cohort study exploring the effects of notched proton beams to date. Future prospective randomised studies would be required to ascertain causality; however, with no clear evidence of disadvantage in terms of tumour control with a notch, this will require large numbers and be difficult to design and recruit.

## 6. Conclusions

In summary, our findings demonstrate that notched proton beam therapy may offer a clinically meaningful reduction in long-term vision loss for tumours located within 3 mm of the optic disc. While not statistically significant, the trend toward slower visual decline and lower complication rates—especially when optic nerve exposure is minimised—supports consideration of this approach in treatment planning. Further validation in prospective, multi-centre studies is warranted.

## Figures and Tables

**Table 1:** Summary of patient characteristics, tumour characteristics for each cohort of patients (notched optic disc, and un-notched); summary of tumour characteristics and staging.

Patient Characteristics	Total	Treated with Notched Beam	Patients with Un-Notched Beam
No. of patients	83	32	51
Age at treatment (median) (mean) (range)			
	59.2 (57.6) (23.3–83.4)	63.0 (59.0) (23.3–83.4)	59.3 (57.7) (33.1–76.2)
Gender			
Male	54 (65.0%)	22 (40.7%)	32 (59.3%)
Female	30 (34.9%)	10 (34.5%)	19 (65.5%)
**Tumour Characteristics**			
Median tumour length (mean) (range)	9.1 (9.7) (2.6–18.1)	9.2 (9.5) (2.6–16.6)	9 (9.9) (5.6–18.1)
Median tumour height (mean) (range)	2.4 (2.63) (0.7–5.9 mm)	2.5 (2.7) (0.7–5.6)	2.3 (2.5) (0.9–5.9)
Median tumour volume (mean) (range)	166.2 mm^3^	165.0 mm^3^	180.8 mm^3^
	(166.12) (2.5–802.4 mm^3^)	(159.2) (2.5–802.4 mm^3^)	(154.6) (16.8–494.9 mm^3^)
Distance from OD (%)			
>2 mm	35 (41.7)	18 (56.3)	16 (34.8)
1.9–1.1 mm	32 (38.1)	13 (40.6)	17 (37.0)
<1 mm	17 (20.2)	1 (3.1)	13 (28.3)
Distance from fovea (%)			
>3 mm	26 (31.0)	8 (25)	17 (37.0)
2.9–0.6 mm	25 (29.8)	8 (25)	14 (30.4)
<0.5 mm	25 (29.8)	14 (43.8)	14 (30.4)
TMN staging (%)			
T1a	23 (27.4)	10 (31.25)	12 (26.1)
T2a	50 (59.5)	17 (53.1)	4 (8.7)
T3a	11 (13.1)	5 (15.6)	5 (10.9)
Mean length of optic nerve treated (mm)	0.5	0.35	1.1

**Table 2:** Shows the Wald statistics of the model.

Predictor	*χ* ^2^	*p*-Value
Optic disc notch (including follow-up interaction *)	4.37	0.1
Follow-up time (including optic disc notch interaction *)	120	<0.0001
* Interaction between follow-up and optic disc notch	3.30	0.07
logMAR0 (nonlinear restricted spline expansion of order 3)	27.2	<0.0001
Age	0.38	0.5
Sex	0.00	1.0
Tumour volume	0.01	0.9
Distance from the fovea	1.51	0.2
Distance from optic disc	1.74	0.2
Tumour stage	3.86	0.1
Radiation retinopathy	1.18	0.3
Optic neuropathy	1.57	0.2
OCT radiation maculopathy	0.67	0.4
Percentage of the macula receiving the prescribed dose	0.32	0.6
Length of optic nerve treated	0.37	0.5
TOTAL	190	<0.0001

**Table 3:** Trends with a 95% confidence interval and standard errors.

Group	Slope of Trend of Visual Acuity Loss (A Positive Value Inferring Increased Loss)	Standard Error
Non-notched	0.29 [0.22, 0.35]	0.034
Notched	0.19 [0.13, 0.25]	0.030

**Table 4:** Comparison of optic disc notch vs. no notch at 6, 8, and 10 years. The estimates are negative, demonstrating there is reduced loss in visual acuity with a notch for the optic disc compared to no notch.

Relative logMAR Difference	Estimate	*Z*	*p*-Value
Optic disc notch—no notch (at 6 years)	−0.35 [−0.80, 0.099]	−1.53	0.13
Optic disc notch—no notch (at 8 years)	−0.47 [−1.0, 0.076]	−1.69	0.092
Optic disc notch—no notch (at 10 years)	−0.57 [−1.2, 0.059]	−1.78	0.076

**Table 5:** Summary of secondary outcomes for each cohort.

Secondary Outcomes	Total	No. Patients with Notched OD (n = 32)	No. Patients with Un-Notched (n = 52)	Odds Ratios of Complications by Beam Notch
Recurrence	3 (3.8%)	1 (3.1%)	2 (4.3%)	0.81
Enucleation	5 (6.0%)	2 (6.3%)	2 (4.3%)	1.67
Radiation retinopathy	43 (51.2%)	13 (40.6%)	28 (60.9%)	0.38
Vitreous haemorrhage	8 (9.5%)	1 (1.2%)	6 (7.1%)	0.25

**Table 6:** Wald statistics and odds ratios (with a 95% confidence interval) of association of optic disc neuropathy with length of optic nerve, optic disc notch, and distance of tumour from optic disc.

	$\mathrm{Wald}\;\chi^{2}$ (1)	*p*-Value	Odds Ratio (95% CI)
Length of optic nerve treated	1.6	0.21	2.2 [0.65, 7.2]
Optic disc notch	0.002	0.96	1.0 [0.34, 3.1]
Distance of tumour from optic disc	1.7	0.19	0.45 [0.14, 1.5]

**Figure A1:**
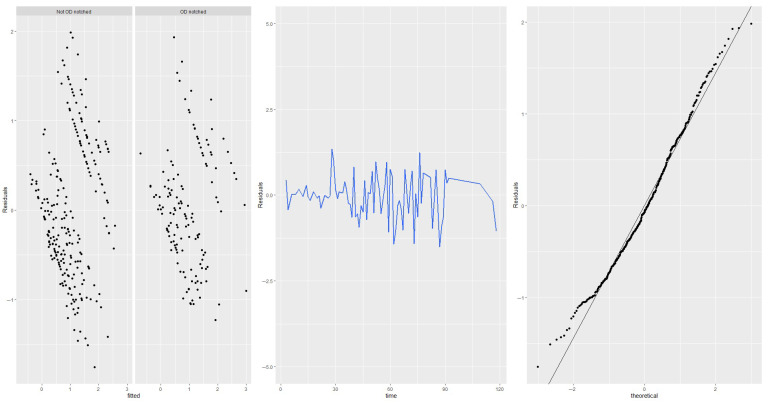
Residual plots.

**Figure A3:**
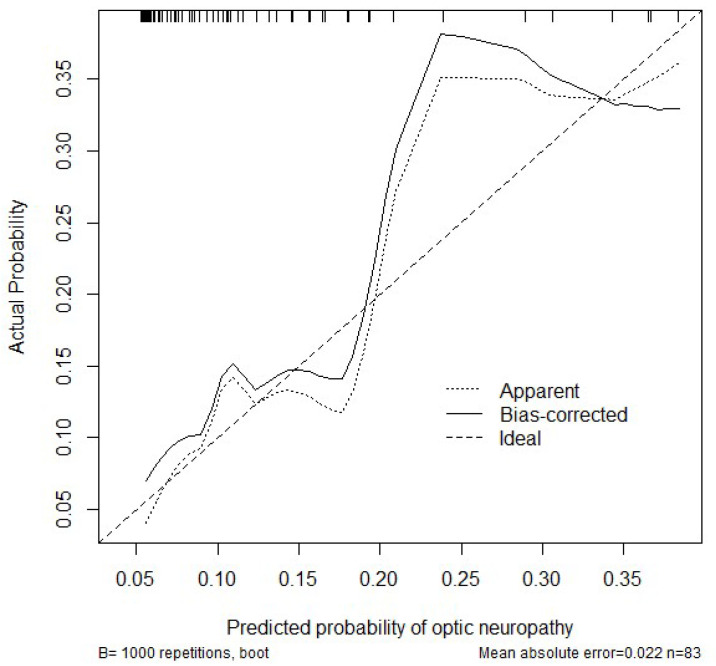
Calibration of the logistic regression model.

## Data Availability

Data set is available upon request from the authors.

## Appendix A

### Appendix A.1. Residuals

Residual plots were used to check for the absence of trends in central tendency and in variability. In Figure A1 we plotted, from left to right: the prediction residuals by fitted values (stratified by notch); the prediction residuals by time; and the distribution of residuals vs. the theoretical distribution. No alarming patterns are evident in any of the plots.

### Appendix A.2. Correlation and Variance Parameters

Table A1 shows the estimated correlation and variance parameters with an approximate 95% confidence interval.

**Table A1:** Correlation and variance parameters.

Parameter	Value [95% Confidence Interval]
Exponential correlation structure: range	3.9 [2.4, 6.4]
Exponential correlation structure: nugget	0.17 [0.092, 0.29]
Variance function: optic disc notch indicator	0.74 [0.63, 0.87]
Residual standard error	0.85 [0.75, 0.97]

In Figure A2 the correlation and variance structures are validated by estimating the semivariogram. The empirical variogram is largely in agreement with the pattern indicated by the exponential correlation and heteroscedastic variance.

## Appendix B

Bootstrap (using 1000 repetitions) has been used to estimate the calibration performance of the model. The graph in Figure A3 shows that the model tends to underestimate the probability of optic neuropathy. The rug plot shows that the observations are quite sparse (corresponding to the low prevalence of optic neuropathy).
